# Genesis of Nanogalvanic Corrosion Revealed in Pearlitic Steel

**DOI:** 10.1021/acs.nanolett.2c02122

**Published:** 2022-09-01

**Authors:** Steven C. Hayden, Claire Chisholm, Shannon L. Eichmann, Rachael Grudt, Gerald S. Frankel, Brian Hanna, Tatiana Headrick, Katherine L. Jungjohann

**Affiliations:** † Aramco Research Center − Boston, Aramco Americas, Cambridge, Massachusetts 02139, United States; ‡ Sandia National Laboratories, Center for Integrated Nanotechnologies, Albuquerque, New Mexico 87185, United States; § Fontana Corrosion Center, Ohio State University, Columbus, Ohio 43210, United States

**Keywords:** Nanogalvanic corrosion, in situ STEM, liquid-cell STEM, nanoscale interface, steel corrosion

## Abstract

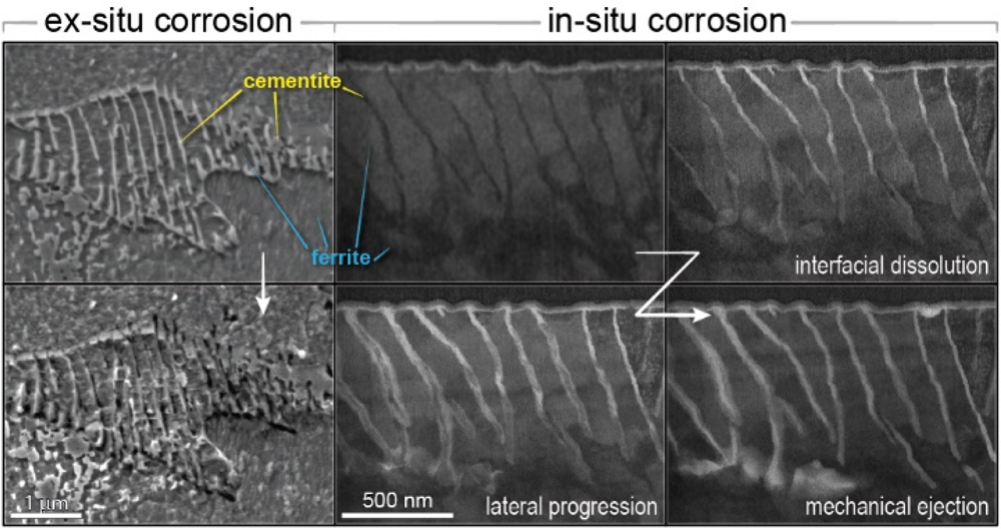

Nanoscale, localized corrosion underpins billions of dollars in damage and material costs each year; however, the processes responsible have remained elusive due to the complexity of studying degradative material behavior at nanoscale liquid–solid interfaces. Recent improvements to liquid cell scanning/transmission electron microscopy and associated techniques enable this first look at the nanogalvanic corrosion processes underlying this widespread damage. Nanogalvanic corrosion is observed to initiate at the near-surface ferrite/cementite phase interfaces that typify carbon steel. In minutes, the corrosion front delves deeper into the material, claiming a thin layer of ferrite around all exposed phase boundaries before progressing laterally, converting the ferrite to corrosion product normal to each buried cementite grain. Over the following few minutes, the corrosion product that lines each cementite grain undergoes a volumetric expansion, creating a lateral wedging force that mechanically ejects the cementite grains from their grooves and leaves behind percolation channels into the steel substructure.

With global infrastructure so heavily supported by low-carbon steel, failures caused by corrosion regularly result in such catastrophic damage to the environment, to the global economy, and to human communities that an estimated 7% of the global GDP is spent preventing, combatting, or paying for the effects of corrosion.^1^ Uniform corrosion, gradual degradation of the entire steel surface, is well-understood. However, localized corrosion, spot degradation that compromises individual points of the steel surface, is far more dangerous because unpredictable, localized nanoscale corrosion phenomena can rapidly evolve into micro- and macroscale holes and crack-initiation sites, even under relatively benign conditions.^2^ Research on localized corrosion began at the macroscale (via electrochemical, weight loss, etc.)^7,8^ and has been extensively conducted at the microscale (e.g., via atomic force and scanning electron microscopy);^3−7^ however, these surface analytical techniques lack the ability to probe processes in situ and at high resolution. Consequently, the ongoing challenge has been to find and observe corrosion initiation on the scale at which it occurs: the nanoscale.^12,13^

Recently published improvements to liquid-cell scanning/transmission electron microscopy (LC-S/TEM)^8−11^ have made it possible to study nanoscale corrosion in real time and to create comprehensive maps of the nano/microstructure, crystal orientation, and elemental composition of steel samples both before and after corrosion.^12,13^ We previously employed this suite of in situ S/TEM and conjoined analytical techniques to identify the nanoscale initiation site for localized corrosion in low-carbon steel: a singular triple junction between a cementite and two ferrite grains.^13^ This work revealed the critical role of the phase interface in the initiation of accelerated, localized corrosion in the immediate region; however, these results were limited to a singular grain boundary between an isolated cementite particle and its neighboring ferrite grains.

Given the critical role of the phase interface implied by our previous work, here we turn our examination to nanoscale corrosion of an entire pearlite colony, a common grain structure in low-carbon steel composed of alternating, interdigitated plates of ferrite and cementite. We begin by observing corrosion in discrete pearlite colonies at the microscale, using the two ex situ methods traditionally employed to study corrosion: scanning electron microscopy and atomic force microscopy (SEM and AFM). We expose segments of pipeline steel to a flowing aqueous corrosion solution (6 μM CO_2_, 281 μM O_2_, 2.78 μM Na_2_SO_4_, pH 6.1, at 10 mL/min) at room temperature and pressure, and then use SEM-coupled energy dispersive X-ray spectroscopy (SEM-EDS) and AFM to observe the composition/morphology and the topography of the localized cementite grains. By taking SEM-EDS images of a pearlite colony before (Figure 1a) and after (Figure 1c) exposure to the corrosive solution, we are able to use the difference image (Figure 1b) to highlight the changes between the time points in the micrographs, showing that the skeletal cementite grains are lost from the pearlite colony over the course of the experiment and highlighting the decreased contrast in Figure 1c, which indicates material loss that overlaps the former positions of the cementite grains and the original pearlite colony boundary. Repetitions of this experiment with shorter time exposures reveal no change in the contrast of the pearlite colonies up to the point where the cementite grains are absent from the image (between 30 and 40 min liquid exposure).

**Figure 1:**
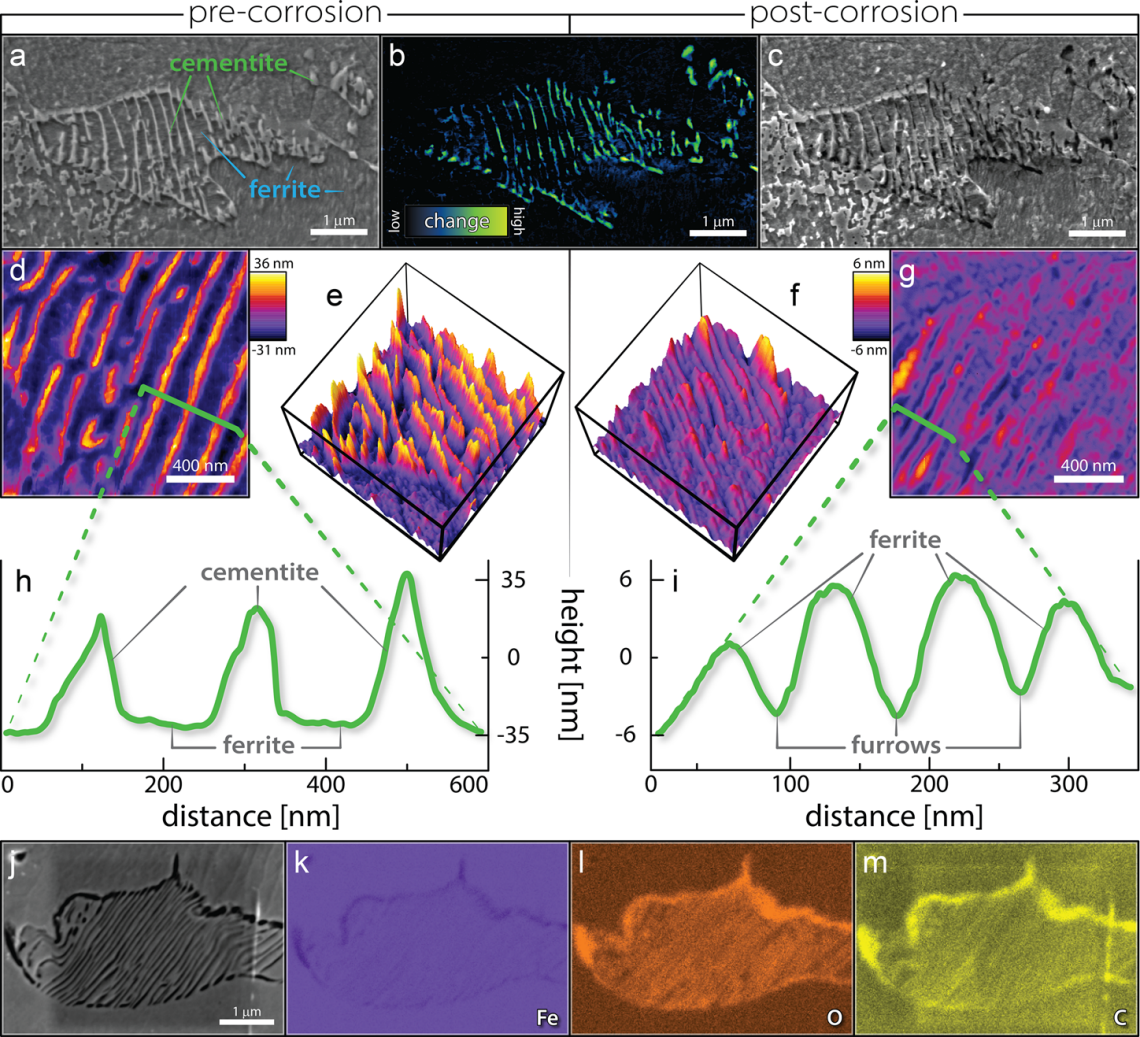
Microscale, ex situ investigation of intragranular pearlite corrosion. A pearlite grain, consisting of alternating plates of ferrite and cementite is shown via SEM micrograph both (a) before and (c) after exposure to the corrosive solution. (b) Difference image highlights that the change between the two micrographs centers almost entirely in the regions where cementite was present in the original pearlite colony. (d–i) AFM was used to confirm that (d, e) the cementite plates visible in the precorroded sample appear to be (f, g) physically absent in the corroded sample, as evidenced in (h, i) the height profiles for the (h) pristine and (i) corroded samples. (j) A different corroded pearlite grain is shown in the SEM micrograph, followed by (k–m) elemental composition maps, in which relative elemental abundance is indicated by color intensity and shows both (k) depleted iron and enhanced corrosion products, (l) oxygen, and (m) carbon, overlapping the regions where the cementite was previously located.

To better understand these topographical changes, we employ AFM to trace the surface topology (Figure 1d–i), which corroborates the observations gleaned from the SEM micrographs. The cementite grains are initially partially raised relative to the surrounding ferrite matrix due to preferential removal of the softer ferrite material during polishing. Following corrosion, AFM height profiles show that the peaks where cementite grains were present in the pristine sample (Figure 1d,e,h) become hollow furrows after corrosion initiation (Figure 1f,g,i). EDS analysis (Figure 1j–m) shows postcorrosion scale formation caused by the depletion of iron (Figure 1k) and a corresponding increase of oxygen (Figure 1l) and carbon (Figure 1m) in the furrowed regions. However, these microscale observations do not provide a mechanistic interpretation because, while ex situ experimentation clearly shows the loss of cementite grains, the fundamental concepts of galvanic corrosion would predict the loss of lower-potential ferrite and the preservation of the higher-potential cementite due to cathodic protection (see the discussion on galvanic corrosion in the Supporting Information).^2,14^ Therefore, we turned to in situ nanoscale observation to reconcile this conflicting observation with predicted mechanisms.

To uncover the nanoscale mechanistic rationale behind this unexplained corrosion phenomenon, we employed the workflow developed in our previous study:^13^ extensive microstructural precharacterization followed by in situ liquid cell STEM to observe corrosion progression in real time. We extract a cross-section of a pearlite colony from the surface of a mechanically polished steel pipe using a focused ion beam (FIB) in an SEM. The resultant cross-sectional slab is ∼150 nm in thickness and captures the near-surface microstructure of the former steel pipe. Figure 2 shows the side view of this slab, where the top of the sample image correlates with the surface of the bulk pipeline steel sample. At the top, two distinct layers of deposited material are visible (see schematic in Figure 2b): a layer primarily composed of amorphous carbon, in physical contact with the former pipe surface, and a layer primarily composed of platinum, on top of the amorphous carbon layer.^15^ These layers are deposited during the standard FIB extraction process, but their chemistry becomes important in the data analysis (*vide infra*). Figure 2c shows the pearlite cross-section mapped via precession electron diffraction (PED), confirming that the orientation of the ferrite in the pearlite colony perpendicular to the slab is largely along the [111] axis and abutted by a region with [101] orientation just below. PED also confirms that the dark stripes are regions of cementite embedded in a ferrite matrix. The cementite grains interdigitate the ferrite matrix, reaching down toward the prior austenite grain boundary (the disordered purple region in Figure 2c). We characterize the cross-section of the pristine structure (via S/TEM and diffraction) and composition (via EDS), before affixing a 30 nm-thick SiN membrane window and loading the sample platform into an in situ liquid-cell holder (Hummingbird Scientific, Lacey, WA) for observation during exposure to the corrosive solution (6 μM CO_2_, 287.5 μM O_2_, 2.78 μM Na_2_SO_4_, pH 6.1, at 2 μL/min) (see the Supporting Information for further notes on STEM data collection and mitigation of the influence of the electron beam).

**Figure 2:**
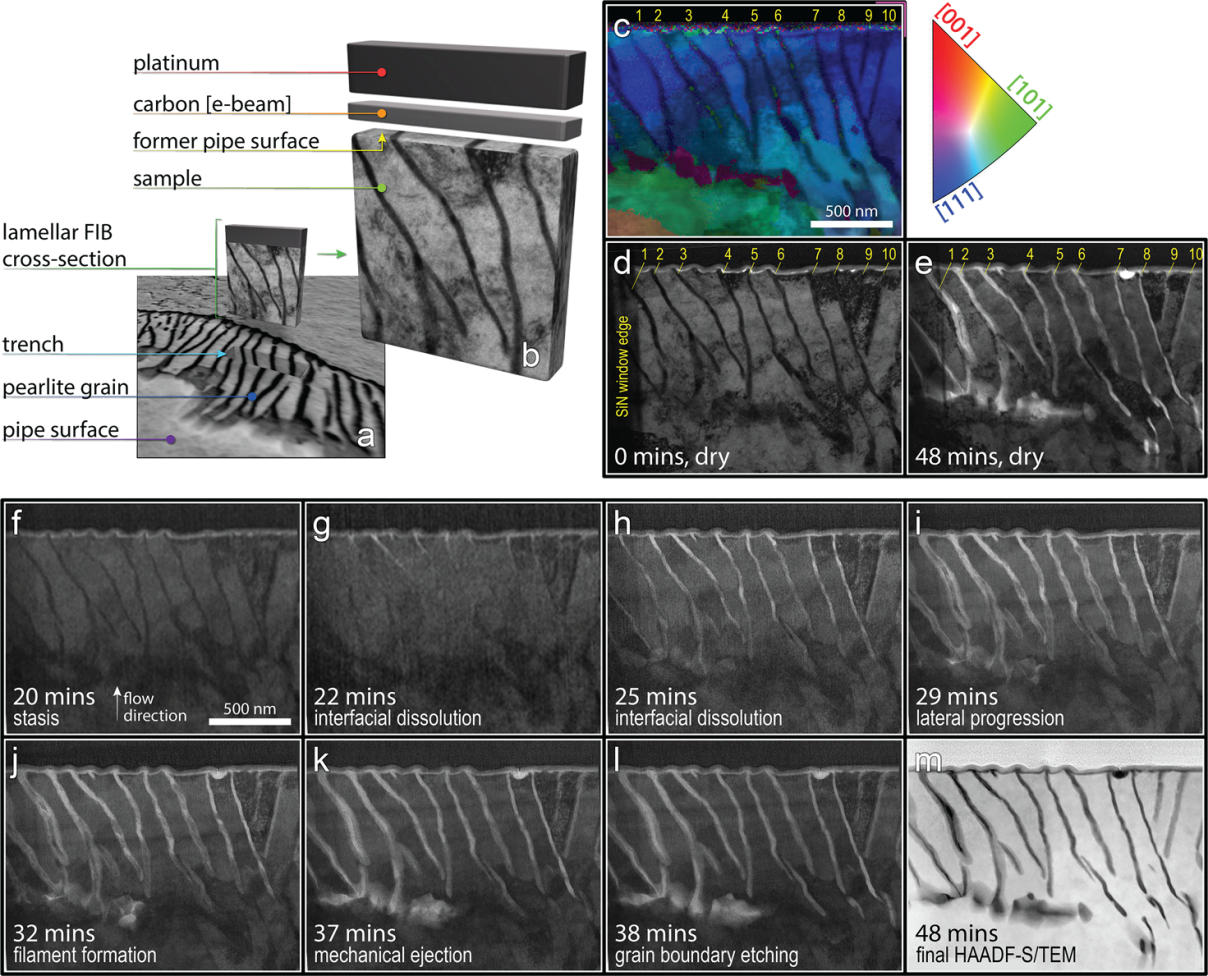
Nanoscale, in situ STEM investigation of intragranular pearlite corrosion. (a) Schematic representation of a pearlite colony with a lamellar FIB section removed such that the interdigitated plates are cross-sectioned (inverted SEM image) and (b) a schematic of the resulting sample sectioned out from the former pipe surface (BF STEM image). (c) PED data indicates the primary crystal planes in the resulting area of interest (blue, [111] for the pearlite colony) as well as crystallographic disorder along the grain boundary (purple). TEM micrographs collected under dry conditions show the sample (d) before and (e) after exposure to the corrosive solution, and yellow numbers indicate the arbitrary grain number assignments for easy reference in the text. (f–l) BF STEM micrographs collected using the liquid cell and flowing solution (2 μL/min) show the sample progressing through various mechanistic stages of corrosion. (f) *Stasis*: During the first 20 min of liquid contact, no change is observed via STEM imaging. (g, h) *Interfacial dissolution*: At 22 min, contrast changes are notable near where the amorphous carbon is in contact with the former pipe surface. The primary contrast changes are localized at the immediate interface between the cementite grains and their ferrite matrix. (i) *Lateral progression*: The corrosion front progresses laterally normal to the cementite/ferrite interface. (j) *Filament formation*: Spontaneous rearrangement of Pt nanocrystals causes a Pt filament to form, which instigates a near-surface pit. (k) *Mechanical ejection*: Volumetric expansion of the corrosion product increases lateral pressure on the cementite grains, forcing them to be mechanically ejected. (l) *Grain boundary etching*: The prior austenite grain boundary along the bottom of the pearlite grain etches steadily from left to right. (m) DF STEM micrograph shows the final structure postcorrosion: empty furrows can be found where the cementite was located previously, and lighter-grey regions of corrosion product are visible.

The in situ STEM results are characterized by a series of sequential events: a period of stasis, then interfacial dissolution, lateral progression out from the phase boundary, followed by mechanical ejection of the cementite grains, and finally etching of the prior austenite grain boundary. Images are collected in bright-field (BF) and dark-field (DF) STEM modes simultaneously to provide optimum contrast for different features in the images; specifically, ferrite and cementite phases are easily distinguished in the BF, whereas voids are easier to visualize in the DF (a video of this data is available as Supporting Information, and snapshots are provided in Figure 2f–m).

## Stasis

The sample is loaded into the in situ holder and prewetted with a droplet of the corrosive solution, then the holder is loaded into the TEM. Static liquid contact lasts approximately 8 min, then liquid flow starts (at 2 μL/min). The first wet image is collected 6 min later (after 14 total minutes of liquid exposure). Compared to the precharacterized sample, no contrast changes are observed for the first 20 min of total liquid exposure (Figure 2f), likely due to the presence of a passivating oxide layer on the sample surface.

## Interfacial Dissolution

**Figure 3:**
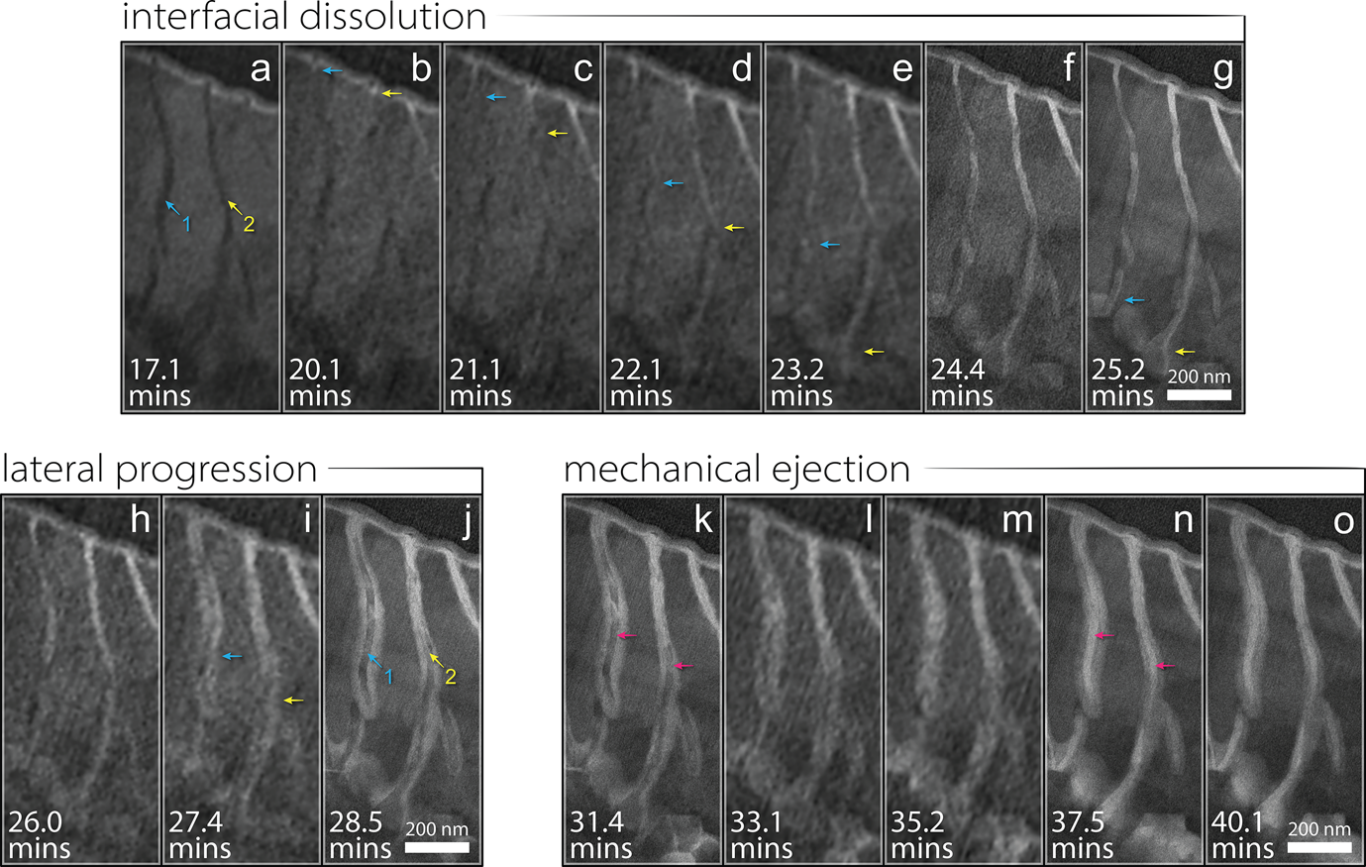
In situ, nanoscale corrosion: focus on cementite grains 1 and 2. (a–o) BF STEM micrographs collected under liquid flow show (b) initiation of accelerated corrosion at the ferrite/cementite grain interface near the former steel pipe surface. (c–g) Yellow and blue arrows track the corrosion as it progresses down, claiming the interfacial regions over about 5 min. Once the ferrite/cementite interface has been claimed, (h, i) the nanoscale corrosion front progresses laterally out from the corroded interface into the surrounding ferrite material. (j) Grains 1 and 2 are still visible in their grooves following this lateral progression. (k–n) Between 31.4 and 37.5 min, lateral pressure from the volumetrically expanding corrosion product lining the trenches forces the cementite grains to be ejected from their grooves. (o) Accelerated nanoscale corrosion is halted in the local region following cementite grain ejection.

Initiation of the corrosion process is first observed at 20 min (Figure 3b) at the top edge of the sample, near the platinum layer, in physical contact with the amorphous carbon layer and the bridging electrolyte, where the local electrochemical environment facilitates initiation.

Initiation of this phase of the attack begins at the former pipe surface, which is in contact with a layer of amorphous carbon that separates the steel from the Pt layer. Both the amorphous carbon and Pt layers were deposited onto the former pipe surface to shield the steel from the ion beam used to extract the sample. The amorphous carbon may attenuate the galvanic interaction between the steel and Pt (vide infra, Pt Filament Formation section), but initiation is still favored in this region due to the proximity of the enhanced cathode provided by the Pt/C construct.

Following initiation at the former pipe surface, interfacial dissolution propagates down the length of the cementite/ferrite phase boundaries, away from the top of the sample, liberating some near-surface fragments of cementite in the process. The corrosion front progresses down into the body of the pearlite colony, claiming the entire interface between the cementite digits and their surrounding ferrite matrix and replacing this interface with corrosion product. Some cementite grains that were fragmented near the surface (likely during sample polishing) are liberated during this period (Figure 2, near-surface parts of grains 4, 5, 7, 8, and Figure 3b); however, the majority of the more deeply buried grains are still visible in their grooves following dissolution of the highly susceptible interfacial region at the immediate cementite/ferrite phase boundary, as resolved in grains 1 and 2 in Figure 3. Note that the cementite grain is still visible in the groove both immediately following interfacial dissolution (Figure 3g) and at the end of the next phase (Figure 3j).

## Lateral Progression

After only 25 min of exposure, the interfacial phase boundaries (the most electrochemically susceptible regions) are corroded away (Figure 3g). At 26 min, the corrosion front begins to progress laterally into the surrounding ferrite (normal to the cementite grains and in the plane of the sample), and lateral progression is completed by 28 min. The lateral front progression is most readily resolved around grains of intact cementite (Figure 3j). However, lateral progression is much less pronounced in empty regions of the furrows, such as the top of grain 2 (Figure 3), which are created when cementite surface fragments are dislodged during interfacial dissolution. This contrast (for our purposes) reconciles the observations because it strongly suggests that the nanoscale corrosion product allows a nanogalvanic corrosion mechanism to progress the corrosion front as cathodic cementite and anodic ferrite form a nanoscale battery when in contact with the aqueous medium.^14^

## Mechanical Ejection

**Figure 4:**
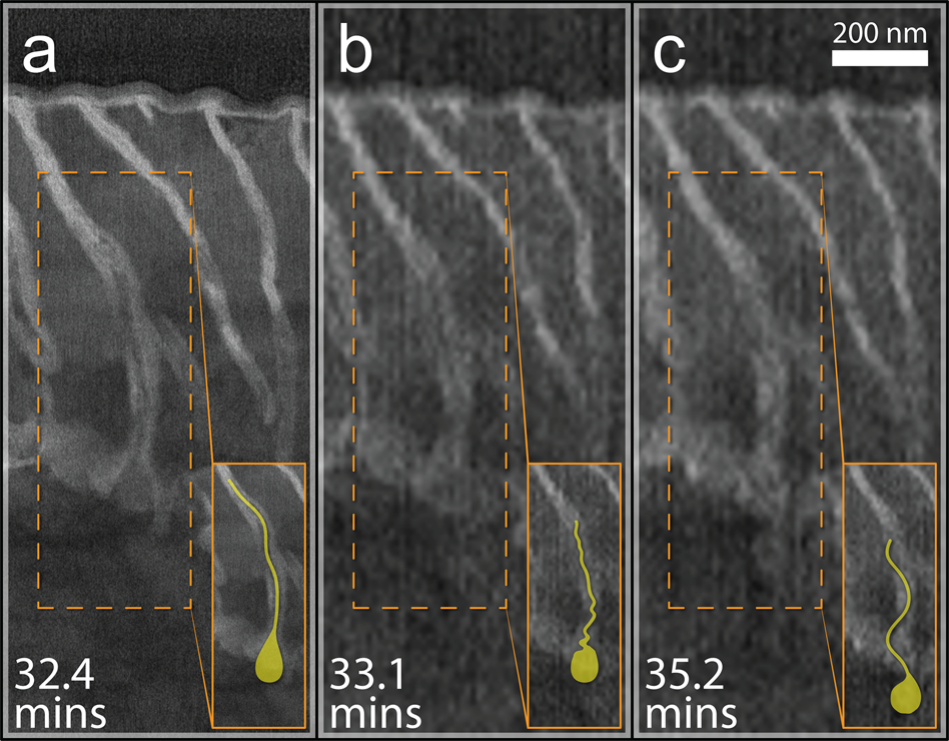
BF STEM micrographs revealing the expulsion, redeposition, and washing away of cementite grain #2 from the pearlite colony. (a) At 32.4 min, the tadpole-shaped grain is still visible in its groove surrounded by lighter-grey corrosion product. (b) At 33.1 min, the head of the tadpole-shape is still embedded in its original position, but the tail has popped out of its groove and deformed. (c) At 35.2 min, the grain is visible as it briefly redeposits on the sample surface before being washed away. These in situ results confirm that the cementite is not being claimed by direct corrosion but rather is mechanically ejected from its matrix. Insets in each panel provide a highlighted view of the grain in question.

From 28 to 35 min, we observe no further changes in contrast. Between 33 and 37 min, the porous-matrix corrosion-product layer surrounding the remaining cementite grains expands, creating a lateral wedging force (elemental compositional analysis of corrosion product in Supporting Information Figures S3 and S4) driven by a large volume ratio of oxide/metal (the Pilling Bedworth ratio). As the corrosion product expands, the cementite grains are mechanically ejected from their grooves (Figure 3k–o). Figure 4a shows cementite grain #2 being mechanically ejected from the groove (Figure 4a), briefly redepositing on the sample surface (Figure 4b), then being washed away (Figure 4c).

## Grain Boundary Etching

At 29 min of liquid exposure, interfacial dissolution and lateral progression have claimed a significant portion of the phase boundaries within the pearlite colony, allowing the electrolyte to penetrate the inner structure (Figure 2m) and contact the prior austenite grain boundary on multiple sides, accelerating corrosion into nonpearlite regions. The grain boundary is steadily converted to corrosion product over the remaining ∼20 min of the experiment (Figure 2j–m).

The experiment is concluded after 48 min of liquid exposure, when the pearlite colony displays a thoroughly compromised structure: cementite grains ejected, remaining structure permeated by void channels, ferrite converted to a mechanically brittle corrosion product, and a severely etched prior-austenite grain boundary region (Figure 2e). Mapped back to the SEM and AFM data presented earlier, the extensive structural damage witnessed in this pearlite grain strongly suggests that continued exposure to the corrosion solution or mechanical perturbations in the region will result in the loss of the entire grain structure.

## Platinum Filament Formation

**Figure 5:**
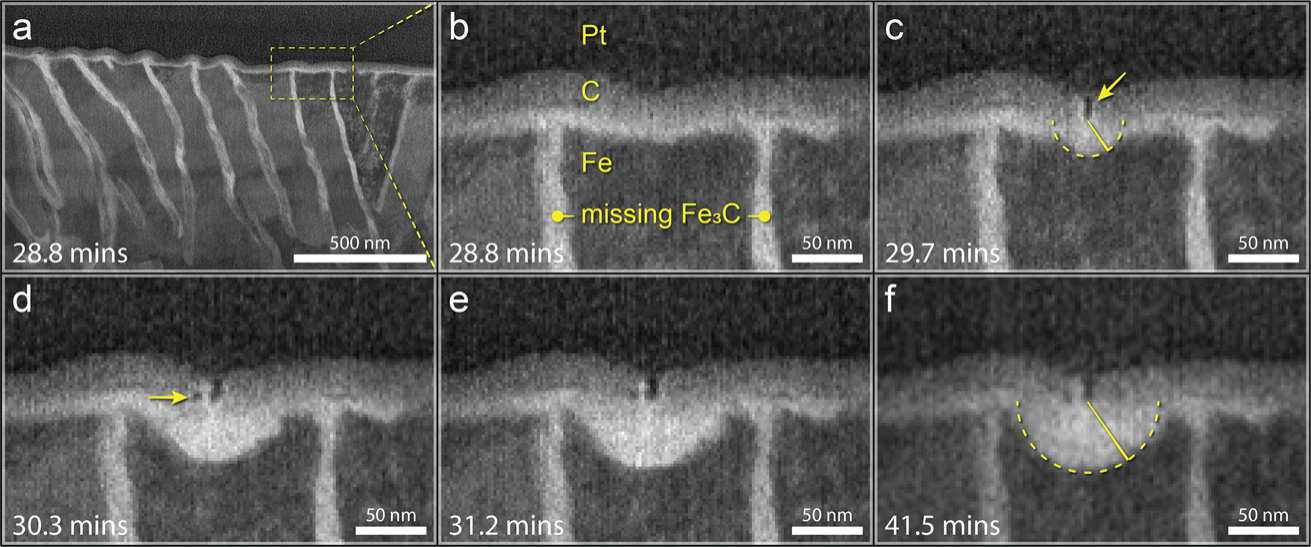
Spontaneous platinum nanofilament formation. (a–f) In situ STEM images depict the region near the former pipe surface where spontaneous formation of a platinum nanofilament is observed. (b) Prior to Pt nanofilament formation, the region is characterized by distinct layers that appear intact. The bright layer between the carbon and metallic steel structure has the same contrast as the corrosion-product-filled prior-cementite region and is likely the oxide layer formed from air exposure on the polished steel surface. (c) At 29.7 min exposure to the corrosion solution, spontaneous rearrangement of Pt nanoparticles in the Pt/C layer leads to the development of a Pt nanofilament that bifurcates the electron-beam-deposited carbon region and electrically bridges the Pt to the ferrite layer; immediate corrosion in the local area is observed radially out from the tip of the nanofilament. (d) At 30.3 min exposure, the hemispherical pit further enlarges, and another lone Pt nanocrystal is observed in the electron-beam carbon layer (yellow arrow). (f) By 31.2 min, the effects of the Pt nanofilament attenuate, and the hemispherical pit is not observed to grow significantly larger through the end of the experiment at 41.5 min.

During this process, we observe an unanticipated phenomenon that requires further investigation (Figure 5). At around 29.7 min, a platinum filament penetrates the amorphous carbon region, thereby creating an intimate connection between the platinum and the ferrite (dark spike, Figure 5c) and instigating accelerated corrosion of the ferrite in the local area. The ferrite corrosion is accelerated radially from the tip of the platinum nanofilament to 49.6 nm. Previous work has shown that the FIB-deposited platinum layer is composed of very small nanocrystals of platinum (ca. 2 nm in diameter) dispersed in a small amount of amorphous carbon.^16^ Given this composition, it is likely that the labile nanocrystals are electrochemically driven to rearrange into the observed filament, which then protrudes through the amorphous carbon layer.^17^ Subsequent frames show the growth of a hemispherical pit (Figure 5c–f) as well as a particle of platinum (small dark circle, Figure 5d) that has broken away from the nanofilament tip but is still encased by the remodeled carbon layer (Figure 5d). At additional time points, the nanofilament seems to further resemble a string of small platinum nanocrystals (Figure 5e).

Spontaneous formation of this nanosized platinum filament is intriguing in its own right,^18,19^ but the response of the ferrite to the sudden introduction of Pt into its local environment also provides fortuitous insight into the ability of the platinum to drive nanogalvanic corrosion of ferrite. The filament breach of the amorphous carbon layer brings Pt closer to the ferrite grain beneath it. The decreased ohmic potential drop results in radial nanogalvanic attack of the ferrite grain, affecting the local electrochemical environment by ionic bridging through the electrolyte to directly impact the ferrite.

The final radius of the hemispherical void (49.6 nm) is greater than the gap separating the platinum matrix from the ferrite sample surface (34–41 nm). This informs our analysis of the interfacial etching initiation discussed above: while the nanogalvanic interaction of Pt/C and ferrite create the driving force to initiate the attack at the cementite/ferrite interface near the top edge of the sample as described above, the range of this interaction is limited. We suggest that based on these findings with the Pt filament, the presence of the Pt/C construct at the top of the sample facilitates accelerated corrosion only in the top ∼10 nm of the sample (50 nm void −40 nm gap = 10 nm). The subsequent progress of the corrosion attack downward to the prior austenite grain boundary resulted primarily from the nanogalvanic interaction of cementite and ferrite. The noncausal nature of the Pt is also supported by both our previous work^13^ and the bulk results in Figure 1, in which interfacial corrosion and cementite ejection, respectively, are observed in the absence of Pt.

Given these nanoscale phenomena gleaned from in situ STEM, we can now rationally reinterpret our original microscale observations made via SEM and AFM at the microscale. Using DF STEM (which provides mass–thickness contrast), we clearly resolve ferrite conversion into corrosion product along the length of the cementite interfaces in the pearlite grain. However, traditional SEM detectors are almost entirely sensitive to topology, with size scale limitations that would impede observation of this thin (<2 nm) corrosion product layer and with much less sensitivity to elemental composition. As a result, in the SEM micrograph, the ferrite is not visually discernible from the oxidized corrosion product. Since ferrite dissolution occurs simultaneously with corrosion product formation, the SEM technique resolved no change in the system up until the point where the cementite was ejected, making it seem as if the higher-potential cementite corrodes before the lower-potential ferrite. Considering these in situ nanoscale STEM results, the microscale SEM-EDS data (Figure 1a–c) showing corrosion-product-lined trenches can be better understood: scale formation within the trenches at the phase interfaces contributes to the ejection of the cementite grains. Given that context, the EDS maps correctly show high carbon and oxygen content (Figure 1j–m) lining the trenches due to the vertical nature of the corrosion-product-lined trenches and the resulting larger sampling volume (several microns subsurface) relative to the former pipe surface.

In summary, we used nanoscale in situ STEM to reinterpret puzzling microscale corrosion data and reveal the actual mechanics of low-carbon steel corrosion initiation. We show that corrosion originates at the phase interfaces at the surface of pearlite structures. The initial corrosion compromises the surface oxide film, then nanogalvanic mechanisms drive corrosion along the ferrite–cementite interfaces, resulting in the creation of percolating nanoscale trenches in the local ferrite, which propagate into the internal structure of the pearlite grain. These trenches are invisible at the microscale because the ferrite and corrosion product are indistinguishable in SEM characterization. However, because the corrosion product is volumetrically larger than the ferrite it replaces, its growth causes lateral strain and resultant mechanical ejection of the cementite from the pearlite. (At the microscale, the disappearing cementite makes it seem as if the higher-potential cementite corrodes before the lower-potential ferrite, which would be counter to the fundamental understanding of corrosion.) The removal of the cementite embrittles the entire pearlite colony, leaving it susceptible to strain-induced collapse. Additionally, the corroded trenches create transport pathways for the electrolyte to compromise the prior austenite grain boundaries and will ultimately spread corrosion to deeper and deeper subsurface pearlite colonies, which can eventually perforate the steel on the macroscale.

The susceptibility of the ferrite/cementite interface to this type of corrosive mechanism is a distressing vulnerability in one of the most common building materials on earth. However, identification of this mechanism should enable the development of protective measures to impede the progression of corrosion, either by passivating the interfaces themselves or by targeting the site of this interfacial mechanism to mitigate strain-induced collapse. Additionally, these findings could change how we understand steel mechanics and corrosion resistance to enable smarter steel manufacturing for the minimization of pearlite grains at steel surfaces.

## Supporting Information Available

Complete in situ BF STEM data frame set; EDS and DF-STEM maps; discussion of S/TEM data collection and possible effect of the electron beam; discussion of galvanic corrosion basics (PDF)
Video of in situ nanogalvanic corrosion (MPG)

## Supplementary Material

nl2c02122_si_001.pdf

nl2c02122_si_002.mpg
