# Supplementary material for: Genesis of Nanogalvanic Corrosion Revealed in Pearlitic Steel
Source: Nano Lett. 2022 Sep 1;22(17):7087–93. doi: 10.1021/acs.nanolett.2c02122 (PMC9479139; doi:10.1021/acs.nanolett.2c02122)
Supplement: Supplementary file 1 — nl2c02122_si_001.pdf [file nl2c02122_si_001.pdf]

Genesis of Nanogalvanic Corrosion Revealed in Pearlitic Steel

Steven C. Hayden,[1†] Claire Chisholm,[2] Shannon L. Eichmann,[1††] Rachael Grudt,[1] Gerald S. Frankel,[3] Brian Hanna,[1] Tatiana Headrick,[1] and Katherine Jungjohann[2†]*

[1] Aramco Research Center – Boston, Aramco Americas, Cambridge, MA 02139, USA
[2] Sandia National Laboratories, Center for Integrated Nanotechnologies, Albuquerque, NM 87185, USA
[3] Fontana Corrosion Center, Ohio State University, Columbus, OH 43210, USA

† Current address: Analytical Microscopy and Imaging Science, National Renewable Energy Laboratory, Denver, CO 80401, USA
†† Current address: Aramco Research Center – Houston, Aramco Americas, Houston, TX 77084, USA
*To whom correspondence should be addressed: katherine.jungjohann@nrel.gov

Supplemental Information

Note on Galvanic Corrosion

Galvanic corrosion occurs when dissimilar conducting materials are electrically connected and exposed in the same electrolyte.[1] The rates of the oxidation and reduction reactions that occur on each material are different than in the uncoupled condition and will change depending on the area ratios of the materials and electrolyte conductivity. For two materials, the more active one with a lower potential will corrode at a faster rate than in the absence of galvanic interaction, and the more noble material with a higher potential will dissolve at a slower rate. For a system with more than two materials, the most and least noble components will behave as expected, and the others might exhibit an increase or decrease in corrosion rate.[2]

S/TEM Data Collection

Characterization of the steel sample was performed on a Tecnai F30 TEM operated at 300 kV to image the microstructure and measure the sample thickness. The corroded sample was imaged using BF/DF STEM, and the relative sample thickness, expressed in units of mean free path, were measured in a grid of discrete points based on the Fourier log-ratio EELS technique using a Tridiem 863 Gatan Image Filter.

The influence of the electron beam was mitigated by decreasing the electron fluence over the liquid cell, both in reducing the STEM beam current and by acquiring images with short dwell times over larger areas, as described previously.[3,4] The imaging conditions used were the same as previously reported.[5] The method of blanking the electron beam between STEM image acquisitions and only acquiring a few longer dwell time exposures allowed us to minimize the electron beam impact to the sample. Liquid flow within the cell during the experiment also aided in removing radiolysis products from the imaging region over the specimen. On average, the dwell time was between 2-10 µs per pixel, and images were recorded at 1024 x 1024 pixels per image. Under these conditions, the electron dose over the sample was far below a threshold of 10 $e^-/Å^2*s$.

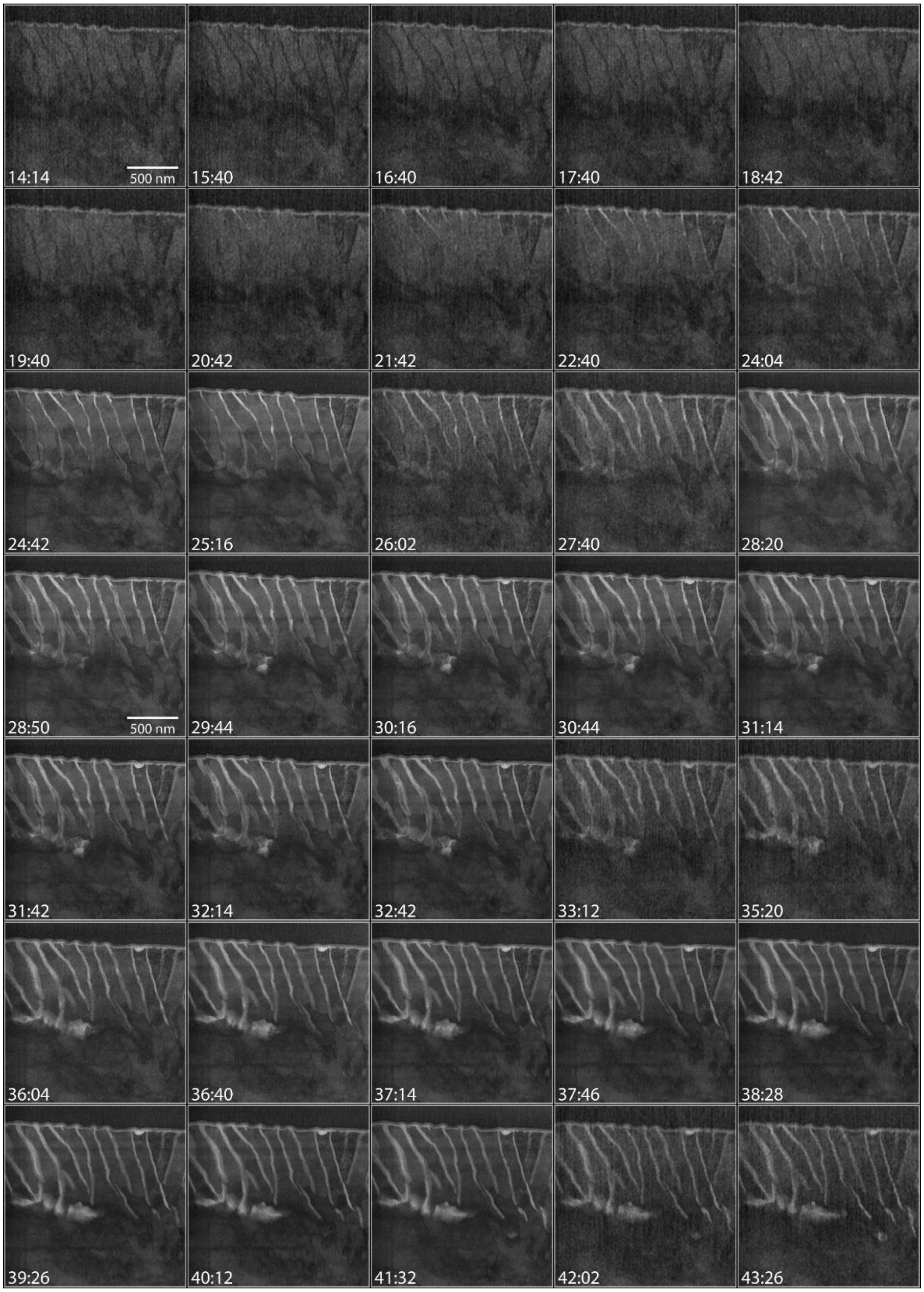

Figure S1. In-situ BF STEM images showing collected frames from experiment start to the end of the in-situ data collection; corresponding timepoints indicated in each frame. Full video is available online.

Elemental Composition (EDS & DF STEM)

Elemental composition maps (via energy dispersive spectroscopy, EDS) and high angle annular dark-field (HAADF) scanning transmission electron microscopy (STEM) micrographs highlight the compositional changes in the corroded sample. Empty furrows are visible where the cementite grains were ejected, and pockets of scale (high in carbon and oxygen content) can be seen lining the furrows in which the cementite persisted through the third phase of corrosion (lateral progression, see main manuscript). Compositional changes are visible as changes in greyscale contrast in the HAADF STEM images. The sample was exposed to air in between HAADF STEM imaging and EDS data acquisition, so corrosion product ratios in select regions are provided to highlight relative differences between the various corroded regions rather than to provide final chemical species identification. The corrosion products observed here match the corrosion products found lining evacuated trenches in the microscale (AFM & SEM) experiments (see main manuscript).

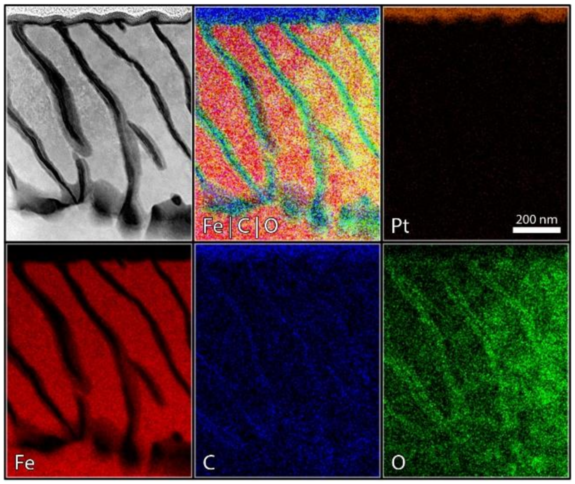

Figure S3. HAADF (top left) and EDS elemental composition maps for the elements indicated in each pane.

Supplemental Information References

1 Jones, D. (1992). Principles and prevention of corrosion. *Macmillan Publishing Company(USA), 1992,*, 568.

2 Shi, L., Song, Y., Zhao, P., Wang, H., Dong, K., Shan, D., & Han, E. H. (2020). Variations of galvanic currents and corrosion forms of 2024/Q235/304 tri-metallic couple with multivariable cathode/anode area ratios: Experiments and modeling. *Electrochimica Acta*, *359*, 136947.

3 Woehl, T. J., Jungjohann, K. L., Evans, J. E., Arslan, I., Ristenpart, W. D., & Browning, N. D. (2013). Experimental procedures to mitigate electron beam induced artifacts during in situ fluid imaging of nanomaterials. *Ultramicroscopy*, *127*, 53-63.

4 Woehl, T. J., Evans, J. E., Arslan, I., Ristenpart, W. D., & Browning, N. D. (2012). Direct in situ determination of the mechanisms controlling nanoparticle nucleation and growth. *ACS nano*, *6*(10), 8599-8610.

5 Hayden, S.C., Chisholm, C., Grudt, R.O., Aguiar, J.A., Mook, W.M., Kotula, P.G., Pilyugina, T.S., Bufford, D.C., Hattar, K., Kucharski, T.J. and Taie, I.M., 2019. Localized corrosion of low-carbon steel at the nanoscale. *npj Materials Degradation*, *3*(1), pp.1-9.
